# Prevalence of Obesity and Malnutrition in Four Cohorts of Very Old Adults, 2000–2017

**DOI:** 10.1007/s12603-022-1820-x

**Published:** 2022-07-06

**Authors:** Maria Burman, C. Hörnsten, J. Öhlin, B. Olofsson, P. Nordström, Y. Gustafson

**Affiliations:** 1 Department of Community Medicine and Rehabilitation, Geriatric Medicine, Umeå University, SE-901 87, Umeå, Sweden; 2 Department of Nursing, Umeå University, Umeå, Sweden

**Keywords:** Body mass index, malnutrition, Mini Nutritional Assessment, obesity, very old adult

## Abstract

**Objectives:**

Investigate trends in the prevalence of obesity and malnutrition among very old adults (age ≥ 85 years) between 2000 and 2017.

**Design, Setting, Participants, Measurements:**

A study with data from the Umeå 85+/Gerontological regional database population-based cohort study of very old adults in northern Sweden. Every 5 years from 2000–2002 to 2015–2017, comprehensive assessments of participants were performed during home visits (N=1602). Body mass index (BMI) classified participants as underweight (<18.5 kg/m^2^), normal weight (18.5–24.9 kg/m^2^), overweight (25.0–29.9 kg/m^2^), and obese (≥30.0 kg/m^2^). Mini Nutritional Assessment (MNA) scores classified participants as malnourished (0 to <17), at risk of malnutrition (17–23.5), and having good nutritional status (24–30). Prevalence and trends were examined using analysis of variance and chi-squared tests, including subgroup analyses of nursing home residents.

**Results:**

Between 2000–2002 and 2015–2017, the mean BMI increased from 24.8± 4.7 to 26.0± 4.7 kg/m^2^. The prevalence of obesity and underweight were 13.4% and 7.6%, respectively, in 2000–2002 and 18.3% and 3.0%, respectively, in 2015–2017. The mean MNA score increased between 2000–2002 and 2010–2012 (from 23.2± 4.7 to 24.2± 3.6), and had decreased (to 23.3± 4.2) by 2015–2017. The prevalence of malnutrition was 12.2%, 5.1%, and 8.7% in 2000–2002, 2010–2012, and 2015–2017, respectively. Subgroup analyses revealed similar BMI and MNA score patterns among nursing home residents.

**Conclusions:**

Among very old adults, the mean BMI and prevalence of obesity seemed to increase between 2000–2002 and 2015–2017. Meanwhile, the nutritional status (according to MNA scores) seemed to improve between 2000–2002 and 2010–2012, it declined by 2015–2017.

## Introduction

**O**besity is associated with numerous diseases and health-related problems in older adults (those aged ≥ 65 years) (1), incurring great health costs (2). However, decreased mortality has been observed in overweight and obese older and very old adults relative to their underweight and normal-weight counterparts (3, 4, 5, 6, 7). The prevalence of obesity among very old (aged ≥ 85 years) adults, is reported at 6.6–10.2% (8, 9). The prevalence of obesity is increasing in older adults (10, 11, 12, 13, 14) and Peralta et al. (15) reported that the prevalence of overweight, but not obesity, in adults aged ≥ 80 years increased between 2005 and 2013.

The reported prevalence of underweight in the very old is 1.2–3.0% (8, 9), and decreased prevalence has been observed among older adults in the last 50 years (16). Underweight is associated with malnutrition, which has severe consequences, including increased mortality (6, 17). Malnutrition and the risk thereof are common among older adults, especially those residing in nursing homes (18, 19). Whether prevalence is changing in very old adults remains uncertain, while a decreased prevalence was seen in nursing home residents (mean age, 86.3 ± 7.7 years) between 1996 and 2010 (20), the prevalence of malnutrition was similar in a population of older adults receiving home care or residing in nursing homes in 2008 and 2013 (19).

The number of very old adults is increasing globally, as well as in Sweden (14, 21). The number of nursing home beds in Sweden has decreased, and the proportion of adults aged ≥ 80 years who lived in nursing homes in Sweden declined from 21.7% in 2001 to 15.6% in 2012 (22). Obesity, underweight and malnutrition are important health issues for very old adults living both in the community and in nursing homes, but little is known about changes in the prevalence of these conditions in this population. The aim of the present study was to investigate whether the prevalence of obesity, underweight, and malnutrition in four cohorts of very old adults in Sweden changed between 2000–2002 and 2015–2017.

## Methods

### Setting and participants

Data for the present study were obtained from the Umeå85+/Gerontological Regional Database (GERDA) study. Individuals living in six municipalities in Västerbotten County, Sweden, were selected from the population register of the Swedish National Tax Board based on age; every other person aged 85 years and every person aged 90 and ≥ 95 years, respectively, was selected to achieve three similarly sized, representative groups. Potential participants received a letter providing information about the study, and were contacted by telephone and asked to provide informed consent to participation 2 weeks later. Data collection began in 2000–2002 (C1) and was repeated every 5 years [in 2005–2007 (C2), 2010–2012 (C3) and 2015–2017 (C4)]. In each period, previous participants and a new representative sample, selected using the identical procedure and inclusion criteria, were asked to participate. Trained assessors (physical therapists, registered nurses, medical students and physicians) performed structured interviews and physical assessments with validated instruments, including the Mini Nutritional Assessment (MNA), in participants' homes. Weight and height were measured with calibrated scales and measuring sticks, and the body mass index (BMI, kilograms per meters squared) was calculated. Participants' medical records were reviewed, and their relatives and/or caregivers contributed additional relevant information as applicable. The Umeå 85+/GERDA study has been described in detail elsewhere (23).

Inclusion criteria for the present study were agreement to participate with a home visit and review of medical records, and documented BMI and MNA score. Participants in C1–C4 were considered as separate cohorts, with survivors assigned to new age groups as appropriate. A flow chart of sample selection is presented in Figure S1 in the Supplementary. This project was conducted according to relevant guidelines and regulations and the study was approved by the Swedish Ethical Review Authority (Dnr 2020-01428).

### Measurements and data collection

The MNA is a screening instrument used to identify risk of malnutrition among older adults. It comprises 18 questions about general health, weight loss, dietary intake, subjective health and nutrition assessments and anthropometric measures (including the BMI). Scores of 0 to <17 indicate malnutrition, those of 17–23.5 indicate a risk of malnutrition and scores of 24–30 indicate good nutritional status (24, 25, 26). Using the World Health Organization's categories, BMIs were used to classify participants as underweight (< 18.5 kg/m^2^), normal weight (18.5–24.9 kg/m^2^), overweight (25.0–29.9 kg/m^2^) and obese (≥ 30.0 kg/m^2^) (27).

The 15-item Geriatric Depression Scale (GDS-15) was used to assess participants' depressive symptoms; scores of 0 to < 5 indicate no depressive symptoms, those of 5 to < 10 indicate mild depression and scores of 10–15 indicate moderate to severe depression (28, 29). To compensate for missing values, GDS-15 scores of participants who answered ≥ 10 questions were imputed according to individual means (total score / total number of questions answered × 15); for participants who answered < 10 questions, GDS-15 scores were designated as missing (30).

The Mini-Mental State Examination (MMSE) was used to evaluate cognitive function; scores range from 0 to 30, with lower scores indicating cognitive impairment (31, 32). Personal activities of daily living (P-ADL) were assessed using the Barthel ADL Index; scores range from 0 to 20, with higher scores indicating higher independence in P-ADL (33). Based on the Katz ADL Index (34), the ADL staircase was used to assess independence in P-ADL and instrumental ADL (I-ADL); results were dichotomised as indicating independence in all 10 activities or dependence in ≥ 1 activity.

Information about diagnoses was collected from participants and their relatives and/or caregivers and from medical records, and/or based on a combination of medical histories, medical records data and the assessments performed for the study. Dementia disorders and depressive disorders were diagnosed according to criteria in the Swedish version of the Diagnostic and Statistical Manual of Mental Disorders, 4th edition, text revision (35). The same geriatrician reviewed all diagnoses in all cohorts. Diagnoses were dichotomised as present (including histories thereof) or absent, except for myocardial infarction, which was coded according to occurrence in the previous year, malignancy, which was coded according to presence in the last 5 years, and urinary tract infection, which was coded according to presence at the time of data collection or in the previous year. Dates of death were collected from participants' medical records. Medications that participants used were classified according to the Anatomical Therapeutic Chemical classification system.

### Statistical analyses

Differences in sex and age between participants and non-participants were analysed using the chi-squared test and the independent samples t test as appropriate. Differences over time were examined using analysis of variance and presented as means with standard deviations for continuous variables, and using chi-squared tests and presented as proportions and percentages for categorical variables. P < 0.05 was considered to indicate significance. Analyses were performed for the four cohorts (C1–C4) and by age group (85, 90 and ≥ 95 years). Sensitivity analyses were performed with the exclusion of individuals who participated more than once (C1, n = 0; C2, n = 115; C3, n = 130; C4, n = 178). Differences among cohorts in associations among the BMI, MNA score and 2-year mortality were analysed using likelihood ratio tests and Cox regression models adjusted for sex and age, with and without interaction terms [BMI/MNA score + cohort vs. BMI/MNA score + cohort + (BMI/MNA score × study cohort)]. The analyses were performed using SPSS (version 25; IBM Corporation, Armonk, NY, USA) and R (version 4.0.3; VR Foundation for Statistical Computing, Vienna, Austria).

## Results

In total, 1602 of 2814 (56.9% [C1, 65.1%; C2, 55.9%; C3, 51.4%; C4, 57.8%]) eligible participants were included in the present study (Figure 1) and mean age did not differ between participants and non-participants (89.9±4.6 and 90.0±4.7 years, respectively), and 60.5% of eligible men and 55.3% of eligible women participated (p = 0.009). Analyses of potential differences between participants and non-participants for each cohort revealed that more eligible men than women participated in the third cohort (60.2% vs. 47.1%, p < 0.001) while the other cohorts showed no sex differences, and no differences in mean age were found between those participating and not in the four separate cohorts.

Participant characteristics are shown by cohort in Table 1 and by age group in Supplementary Tables Table 1, Table 2, Table 3. Several diagnoses and the number of prescribed drugs differed significantly among cohorts, whereas the mean MMSE and Barthel ADL Index scores did not. The proportion of the population living in nursing homes differed among cohorts (p = 0.001); it was 39.7% for the C1 population and 26.6% for the C4 population (Table 1). The proportion of individuals aged ≥ 95 years (22.4% and 30.1% of the whole sample of C1 and C4 populations, respectively [Table 1]) who were living in nursing homes also differed among cohorts (p = 0.038); it was 70.1% for the C1 population and 51.0% for the C4 population (Supplementary Table 3).

**Table 1:** Baseline characteristics of participants, according to cohorts

	**2000–2002 (C1)**	**2005–2007 (C2)**	**2010–2012 (C3)**	**2015–2017 (C4)**	
**Characteristic**	**(n = 343)**	**(n = 342)**	**(n = 409)**	**(n = 508)**	**p**
Women	243 (70.8)	231 (67.5)	253 (61.9)	334 (65.7)	0.069
Age mean (years)	89.5 ± 4.5	90.1 ± 4.4	89.8 ± 4.7	90.2 ± 4.6	0.182
Age range (years)	85–103	84–104	84–105	84–102	
Age group (years)					0.160
85	137 (39.9)	123 (36.0)	150 (36.7)	166 (32.7)	
90	129 (37.6)	129 (37.7)	139 (34.0)	189 (37.2)	
≥95	77 (22.4)	90 (26.3)	120 (29.3)	153 (30.1)	
Living in residential care facilities	136 (39.7)	116 (33.9)	132 (32.3)	135 (26.6)	0.001
> 8 years education (n = 1524)	255 (75.2)	237 (78.0)	289 (73.7)	310 (63.4)	<0.001
Current smoker (n = 1595)	14 (4.1)	11 (3.2)	9 (2.2)	9 (1.8)	0.204
Barthel ADL Index (0–20; n = 1599)	16.2 ± 5.9	16.7 ± 5.0	16.9 ± 4.8	16.5 ± 5.5	0.338
Independence in P-ADL & I-ADL^a^ (n=1599)	79 (23.1)	96 (28.1)	72 (17.6)	100 (19.8)	0.003
GDS-15 score (n = 1486)	3.8 ± 2.7	3.7 ± 2.7	3.5 ± 2.5	3.3 ± 2.5	0.014
MMSE score (n = 1567)	21.8 ± 7.8	21.0 ± 6.8	21.7 ± 6.5	21.8 ± 6.8	0.299
**Diagnoses**					
Constipation	139 (40.5)	168 (49.1)	200 (48.9)	273 (53.7)	0.002
COPD	48 (14.0)	56 (16.4)	84 (20.5)	89 (17.5)	0.120
Dementia disorder	92 (26.8)	114 (33.3)	150 (36.7)	203 (40.0)	0.001
Depressive disorder	93 (27.1)	141 (41.2)	181 (44.3)	208 (40.9)	<0.001
Diabetes mellitus	45 (13.1)	46 (13.5)	80 (19.6)	85 (16.7)	0.049
Diarrhoea	34 (9.9)	45 (13.2)	53 (13.0)	115 (22.6)	<0.001
Heart failure	85 (24.8)	96 (28.1)	154 (37.7)	146 (28.7)	0.001
Hip fracture	70 (20.4)	54 (15.8)	78 (19.1)	92 (18.1)	0.453
Hypertension	189 (55.1)	235 (68.7)	328 (80.2)	404 (79.5)	<0.001
Malignancy^b^	36 (10.5)	31 (9.1)	60 (14.7)	92 (18.1)	<0.001
Myocardial infarction^c^	12 (3.5)	11 (3.2)	10 (2.4)	2 (0.4)	
Parkinson's disease	9 (2.6)	5 (1.5)	2 (0.5)	5 (1.0)	
Stroke	68 (19.8)	75 (21.9)	103 (25.2)	105 (20.7)	0.274
Thyroid disease	40 (11.7)	47 (13.7)	89 (21.8)	108 (21.3)	<0.001
Urinary tract infection^d^	99 (28.9)	92 (26.9)	83 (20.3)	75 (14.8)	<0.001
**Drug prescriptions**					
Number of drugs^e^	6.4 ± 4.4	8.2 ± 5.1	8.6 ± 4.4	8.3 ± 4.6	<0.001
Analgesics	253 (73.8)	264 (77.2)	326 (79.7)	368 (72.4)	0.055
Antidepressants	56 (16.3)	56 (16.4)	82 (20.0)	100 (19.7)	0.359
Cholinesterase inhibitors	7 (2.0)	14 (4.1)	7 (1.7)	14 (2.8)	0.194
Corticosteroids, oral	18 (5.2)	25 (7.3)	16 (3.9)	25 (4.9)	0.212
Diuretics	167 (48.7)	170 (49.7)	230 (56.2)	221 (43.5)	0.002
Drugs for acid-related symptoms	42 (12.2)	77 (22.5)	110 (26.9)	124 (24.4)	<0.001
Insulin	11 (3.2)	8 (2.3)	35 (8.6)	37 (7.3)	<0.001
Laxatives	122 (35.6)	135 (39.5)	155 (37.9)	184 (36.2)	0.696
Mirtazapine	2 (0.6)	11 (3.2)	13 (3.2)	24 (4.7)	
Neuroleptics	28 (8.2)	23 (6.7)	13 (3.2)	18 (3.5)	0.003
Opioids	70 (20.4)	72 (21.1)	64 (15.6)	71 (14.0)	0.016
Oral antihyperglycemics	19 (5.5)	33 (9.6)	36 (8.8)	23 (4.5)	0.008
Paracetamol	144 (42.0)	183 (53.5)	234 (57.2)	299 (58.9)	<0.001
SSRIs	47 (13.7)	47 (13.7)	60 (14.7)	73 (14.4)	0.975
Vitamin B12	87 (25.4)	146 (42.7)	133 (32.5)	154 (30.3)	<0.001


Data are presented as mean ± standard deviation or n (%), unless otherwise indicated. Differences in mean values were examined using one-way analysis of variance. Differences in proportions were analysed using the chi-squared test. a. According to the ADL staircase. b. In the previous 5 years. c. In the previous year. d. At present or in the previous year. e. Regular use and pro re nata. ADL, activities of daily living; C, cohort; COPD, chronic obstructive pulmonary disease; GDS-15, 15-item Geriatric Depression Scale; I, instrumental; MMSE, Mini-Mental State Examination; P, personal; SSRI, selective serotonin reuptake inhibitor.

**Table 2:** Differences in BMI among cohorts

	**2000–2002 (C1)**	**2005–2007 (C2)**	**2010–2012 (C3)**	**2015–2017 (C4)**	**p**
	**(n = 343)**	**(n = 342)**	**(n = 409)**	**(n = 508)**	
**Whole sample**					
Mean BMI	24.8 ± 4.7	24.9 ± 4.1	25.4 ± 4.2	26.0 ± 4.7^a^,^b^	<0.001
BMI categories					0.006
<18.5	26 (7.6)	18 (5.3)	18 (4.4)	15 (3.0)	
18.5–24.9	173 (50.4)	162 (47.4)	187 (45.7)	218 (42.9)	
25.0–29.9	98 (28.6)	121 (35.4)	148 (36.2)	182 (35.8)	
≥30.0	46 (13.4)	41 (12.0)	56 (13.7)	93 (18.3)	
**85 years**					
Mean BMI	25.6 ± 4.2	25.7 ± 4.0	26.2 ± 4.4	27.4 ± 4.7^a^,^b^	0.001
BMI categories					0.112
<18.5	4 (2.9)	3 (2.4)	4 (2.7)	2 (1.2)	
18.5–24.9	72 (52.6)	55 (44.7)	63 (42.0)	57 (34.3)	
25.0–29.9	44 (32.1)	44 (35.8)	54 (36.0)	67 (40.4)	
≥30.0	17 (12.4)	21 (17.1)	29 (19.3)	40 (24.1)	
**90 years**					
Mean BMI	25.0 ± 4.9	24.7 ± 4.0	25.3 ± 4.1	25.7 ± 4.5	0.191^c^
BMI categories					0.227
<18.5	11 (8.5)	5 (3.9)	6 (4.3)	6 (3.2)	
18.5–24.9	53 (41.1)	70 (54.3)	65 (46.8)	83 (43.9)	
25.0–29.9	42 (32.6)	41 (31.8)	48 (34.5)	67 (35.4)	
≥30.0	23 (17.8)	13 (10.1)	20 (14.4)	33 (17.5)	
**≥ 95 years**					
Mean BMI	23.0 ± 4.5	24.3 ± 4.2	24.5 ± 3.7	25.0 ± 4.6^a^	0.008
BMI categories					0.003
<18.5	11 (14.3)	10 (11.1)	8 (6.7)	7 (4.6)	
18.5–24.9	48 (62.3)	37 (41.1)	59 (49.2)	78 (51.0)	
25.0–29.9	12 (15.6)	36 (40.0)	46 (38.3)	48 (31.4)	
≥30.0	6 (7.8)	7 (7.8)	7 (5.8)	20 (13.1)	


Data are presented as mean ± standard deviation or n (%). Differences in mean values were examined using one-way analysis of variance with Bonferroni correction. Differences in proportions were examined using the chi—squared test. Post hoc tests: a. significant difference vs. C1; b. significant difference vs. C2; c. no significant difference. BMI, body mass index (kg/m^2^); C, cohort.

**Table 3:** Differences in MNA score among cohorts

	**2000–2002 (C1)**	**2005–2007 (C2)**	**2010–2012 (C3)**	**2015–2017 (C4)**	**p**
	**(n = 343)**	**(n = 342)**	**(n = 409)**	**(n = 508)**	
**Whole sample**					
Mean MNA score	23.2 ± 4.7	23.7 ± 3.9	24.2 ± 3.6^a^	23.3 ± 4.2^b^	0.002
MNA score categ.					0.004
<17	42 (12.2)	22 (6.4)	21 (5.1)	44 (8.7)	
17.0–23.5	109 (31.8)	126 (36.8)	131 (32.0)	189 (37.2)	
24–30	192 (56.0)	194 (56.7)	257 (62.8)	275 (54.1)	
**85 years**					
Mean MNA score	24.9 ± 3.2	24.8 ± 3.5	25.0 ± 3.2	24.9 ± 3.5	0.991^c^
MNA score categ.					0.792
<17	5 (3.6)	3 (2.4)	3 (2.0)	7 (4.2)	
17.0–23.5	35 (25.5)	34 (27.6)	44 (29.3)	38 (22.9)	
24–30	97 (70.8)	86 (69.9)	103 (68.7)	121 (72.9)	
**90 years**					
Mean MNA score	22.8 ± 4.6	23.6 ± 3.8	24.2 ± 3.4^a^	23.3 ± 4.1	0.039
MNA score categ.					0.074
<17	16 (12.4)	8 (6.2)	5 (3.6)	12 (6.3)	
17.0–23.5	46 (35.7)	53 (41.1)	46 (33.1)	75 (39.7)	
24–30	67 (51.9)	68 (52.7)	88 (63.3)	102 (54.0)	
**≥ 95 years**					
Mean MNA score	20.6 ± 5.7	22.3 ± 4.3	23.1 ± 3.9^a^	21.4 ± 4.2^b^	0.001
MNA score categ.					0.002
<17	21 (27.3)	11 (12.2)	13 (10.8)	25 (16.3)	
17.0–23.5	28 (36.4)	39 (43.3)	41 (34.2)	76 (49.7)	
24–30	28 (36.4)	40 (44.4)	66 (55.0)	52 (34.0)	


Data are presented as mean ± standard deviation or n (%). Differences in mean values were examined using one-way analysis of variance with Bonferroni correction. Differences in proportions were analysed using the chi—squared test. Post-hoc tests: a. significant difference vs. C1; b. significant difference vs. C3; c. no significant difference. MNA, Mini Nutritional Assessment.

Differences in the BMI among cohorts are presented for the whole sample and by age group in Table 2. The mean BMI increased between C1 and C4 in the whole sample (from 24.8 ± 4.7 to 26.0 ± 4.7 kg/m^2^; p < 0.001), and similar changes were seen in all age groups except 90-year-olds. In the whole sample, the prevalence of obesity was 13.4% at C1 and 18.3% at C4. In the most recent cohort (C4), 24.1% of 85-year-olds, 17.5% of 90-year-olds and 13.1% of those aged ≥ 95 years were obese. The prevalence of underweight in the whole sample was 7.6% at C1 and 3.0% at C4 (Table 2).

Differences among cohorts in MNA scores for the whole sample and by age group are presented in Table 3. In the whole sample, the mean MNA score increased between C1 and C3 (from 23.2 ± 4.7 to 24.2 ± 3.6), and decreased (to 23.3 ± 4.2) between C3 and C4 (overall, p = 0.002); similar results were obtained for 90-year-olds and individuals aged ≥ 95 years. Hence, in the whole sample, the mean MNA score did not differ between C1 and C4. In the whole sample, the proportions of participants classified as malnourished were 12.2% at C1, 6.4% at C2, 5.1% at C3 and 8.7% at C4. Similar trends were seen in all age groups, but MNA-based classification differed among cohorts only in the oldest age group (p = 0.002). In the most recent cohort (C4), 4.2% of 85-year-olds, 6.3% of 90-year-olds and 16.3% of those aged ≥ 95 years were classified as malnourished (Table 3).

The results of sensitivity analyses did not differ considerably from those obtained for the whole sample (Supplementary Tables 4–6). The results of analyses performed for the subgroup of nursing home residents are shown in Table 4. The mean BMI increased from C1 and C4 in this whole subgroup (from 24.1 ± 5.3 to 25.8 ± 5.2 kg/m^2^; p = 0.024) and in those aged ≥ 95 years (p = 0.012). The mean MNA score increased between C1 and C3 in this whole subgroup (from 20.1 ± 5.0 to 21.7 ± 3.8) and in those aged ≥ 95 years. This score decreased between C3 and C4 in the whole subgroup (from 21.7 ± 3.8 to 19.5 ± 4.3), in 90-year-olds and in individuals aged ≥ 95 years (Table 4).

**Table 4:** Differences in BMI and MNA score among subcohorts of participants living in nursing homes

	**2000–2002 (C1)**	**2005–2007 (C2)**	**2010–2012 (C3)**	**2015–2017 (C4)**	**p**
**Whole sample**	**(n = 136)**	**(n = 116)**	**(n = 132)**	**(n = 135)**	
Mean BMI	24.1 ± 5.3	24.9 ± 4.3	25.4 ± 4.3	25.8 ± 5.2^a^	0.024
Mean MNA score	20.1 ± 5.0	21.1 ± 3.8	21.7 ± 3.8^a^	19.5 ± 4.3^b^,^c^	<0.001
**85 years**	**(n= 24)**	**(n = 25)**	**(n = 23)**	**(n = 16)**	
Mean BMI	27.4 ± 5.7	25.6 ± 3.7	26.8 ± 5.0	26.7 ± 4.8	0.607^d^
Mean MNA score	22.3 ± 3.3	22.0 ± 4.0	21.8 ± 3.3	19.9 ± 3.5	0.173^d^
**90 years**	**(n= 58)**	**(n = 40)**	**(n = 46)**	**(n = 41)**	
Mean BMI	24.0 ± 5.0	25.1 ± 4.5	25.9 ± 4.4	26.3 ± 5.4	0.096^d^
Mean MNA score	20.5 ± 5.0	20.6 ± 3.3	22.0 ± 3.7	19.5 ± 4.7^c^	0.054
**≥ 95 years**	**(n = 54)**	**(n = 51)**	**(n = 63)**	**(n = 78)**	
Mean BMI	22.7 ± 4.7	24.4 ± 4.3	24.5 ± 3.7	25.4 ± 5.2^a^	0.012
Mean MNA score	18.7 ± 5.3	21.0 ± 4.1	21.5 ± 4.1^a^	19.4 ± 4.3^c^	0.002


Data are presented as mean ± standard deviation. Differences in mean values were examined using one-way analysis of variance with Bonferroni correction. Post -hoc tests: a. significant difference vs. C1; b. significant difference vs. C2; c. significant difference vs. C3; d. no significant difference. BMI, body mass index (kg/m^2^); MNA, Mini Nutritional Assessment.

Two-year mortality data are presented for the whole sample and by BMI and MNA categories in Table 5. In the four cohorts, 27.8–28.6% of individuals died within 2 years. The mortality rate was 16.2–16.7 deaths per 100 person-years in the whole sample, 7.6–12.7 deaths per 100 person-years among obese individuals, 29.9–66.0 deaths per 100 person-years among underweight individuals and 39.4–70.3 deaths per 100 person-years among malnourished individuals (Table 5). Likelihood ratio tests revealed no difference among cohorts in the associations of the BMI and MNA score with 2-year mortality (continuous BMI, p = 0.932; categorical BMI, p = 0.401; continuous MNA, p = 0.126; categorical MNA, p = 0.451).

**Table 5:** Two-year mortality in the whole population and according to BMI and MNA score

	**2-year mortality**	**2000–2002 (C1)**	**2005–2007 (C2)**	**2010–2012 (C3)**	**2015–2017 (C4)**
Whole sample					
	Deaths	97/343 (28.3)	95/342 (27.8)	117/409 (28.6)	142/508 (28.0)
	Deaths/100 person-years	16.5	16.2	16.7	16.7
BMI categ.					
<18.5	Deaths	12/26 (46.2)	13/18 (72.2)	9/18 (50.0)	7/15 (46.7)
	Deaths/100 person-years	29.9	66.0	33.7	38.4
18.5–24.9	Deaths	57/173 (32.9)	43/162 (26.5)	54/187 (28.9)	66/218 (30.3)
	Deaths/100 person-years	20.0	15.5	17.0	18.4
25.0–29.9	Deaths	18/98 (18.4)	30/121 (24.8)	46/148 (31.1)	48/182 (26.4)
	Deaths/100 person-years	10.1	14.0	18.3	15.5
≥30.0	Deaths	10/46 (21.7)	9/41 (22.0)	8/56 (14.3)	21/93 (22.6)
	Deaths/100 person-years	12.2	12.1	7.6	12.7
MNA score categ.					
<17	Deaths	23/42 (54.8)	14/22 (63.6)	17/21 (81.0)	32/44 (72.7)
	Deaths/100 person-years	39.4	48.6	70.3	68.3
17.0–23.5	Deaths	44/109 (40.4)	54/126 (42.9)	52/131 (39.7)	69/189 (36.5)
	Deaths/100 person-years	25.6	27.8	24.8	23.2
24–30	Deaths	30/192 (15.6)	27/194 (13.9)	48/257 (18.7)	41/275 (14.9)
	Deaths/100 person-years	8.4	7.4	10.3	8.1


Data are presented as n(%) or n. BMI,body mass index (kg/m^2^); MNA,Mini Nutritional Assessment.

## Discussion

In this study of very old adults in northern Sweden, the mean BMI increased over the study period, with a corresponding increase in the prevalence of obesity and decline in the prevalence of underweight. The mean MNA score did not differ between C1 and C4, but increased and then declined in the intervening period. The prevalence of malnourishment was greatest at C1, then declined and increased again to a lesser extent over the remaining study period. Similar BMI and MNA results were obtained for the subgroup of nursing home residents. The associations of the BMI and MNA score with 2-year mortality did not differ among the four cohorts.

The increases in the mean BMI and proportion of obese individuals over time contrast with some previously reported results (15), but are in agreement with findings for older adults in general (36, 37). Also, obesity seemed to be more common in 85-year-olds than in those aged ≥ 95 years, supporting previous results that BMI decline in old age (38, 39).

This study also showed that the prevalence of underweight in very old individuals declined, but the mean MNA score did not differ between C1 and C4. Although body weight is associated with nutritional status, our results suggest that factors other than the BMI affect MNA scores in this very old population. The lack of change in nutritional status is in agreement with previously reported results (19). However, the nutritional status of very old individuals improved over the first 10 years of the study period and had declined in the last cohort. These findings have several possible explanations. Direct and indirect effects of certain conditions (e.g. dementia, depression and malignancy), the number of drugs used and ADL dependency appeared to be more common in later cohorts. In addition, the larger proportion of the oldest individuals in the C4 cohort relative to the C1 cohort may have contributed to the greater prevalence of conditions whose prevalence increases with age, such as dementia (40, 41). All of these factors imply that the MNA score would be lower in the later cohorts.

In 2008, a Swedish national quality register (Senior Alert) was launched to prevent (among other things) malnutrition through risk assessments and the implementation of action plans (42, 43). In addition, a national economic stimulus was provided in 2010–2014 to improve the care of older adults in Sweden, including the Senior Alert programme (44). As nutritional screening is associated with a decline in the prevalence of malnutrition in nursing homes (45), these initiatives may have contributed to the improvement of nutritional status, especially among those living in nursing homes, where many the of Senior Alert registration takes place. This potential improvement in nutritional status, however, may have been counteracted by the decline in the availability of nursing home beds and the increased frailty (and thus increased malnutrition risk) of adults living in these facilities over time (46). The present study provides new information about trends in the nutritional status of very old adults; the improvement seen during the first 10 years in this population overall and in nursing home residents is in agreement with previous findings from Sweden (20). However, the reversal of this trend in the latest cohort needs to be investigated further.

Several limitations of this study should be considered. The BMI is an easy-to-use, cost-effective and well-established measure, but it is an indirect measure of body fat and does not consider body constitution (47). Its use for older adults has been questioned (48), as age-related height decreases falsely inflate BMI values, which needs to be considered when interpreting results (49). The MNA is a commonly used and recommended screening instrument for the detection of malnutrition risk in older adults; further assessment, however, is required to definitively diagnose malnutrition (50). This cross-sectional study was performed to investigate whether the prevalence of obesity, underweight and malnutrition according to MNA scores had changed over time; the investigation of potential reasons for these changes was beyond its scope. Clinical and/or diagnostic practice may have changed over the 15-year study period, potentially affecting the reported prevalence of some conditions. However, the same procedure for the collection of information about diagnoses was used at all times, and the same experienced geriatrician evaluated the diagnostic data, thereby limiting variation among cohorts. Other factors than those discussed may also have an effect on the observed time trends, such as socio-economic changes and improved oral health. Differences or lack thereof in the BMI and MNA score between the four cohorts, might also be explained by a variation in the samples. Furthermore, this study was population based, with representative samples of each age group included in all cohorts and the same sample selection and inclusion criteria used in all collection periods. Trained assessors performed evaluations in participants' homes, including nursing homes, and weight and height were measured using calibrated scales and measuring sticks to avoid errors (51).

## Conclusion

In this study of very old adults in northern Sweden, the mean BMI and prevalence of obesity had increased in the last 15 years. The nutritional status (according to MNA scores) improved in the first 10 years in the whole sample and in nursing home residents, but a worrying trend suggesting a worsening nutritional status was seen in the last five years. From a public health perspective, it is important to follow changes in nutritional status in very old adults and explore underlying causes and consequences.

## Electronic Supplementary Material

**Supplementary Figure 1** Flow chart of study participation

Supplementary material, approximately 57.6 KB.
