# Supplementary material for: Prevalence of Obesity and Malnutrition in Four Cohorts of Very Old Adults, 2000–2017
Source: J Nutr Health Aging. 2022 Jul 6;26(7):706–13. doi: 10.1007/s12603-022-1820-x (PMC12876584; doi:10.1007/s12603-022-1820-x)
Supplement: Supplementary file 1 — Supplementary Figure 1 Flow chart of study participation [file mmc1.docx]

**Supplementary Figure 1**

Flow chart of study participation

Flow chart of study participation for the four cohorts with data collection in 2000–2002, 2005–2007, 2010–2012, and in 2015–2017, according to age group (85, 90 and ≥ 95 years). BMI, body mass index; C, cohort; MNA, Mini Nutritional Assessment.
