# Supplementary material for: Prevalence of Obesity and Malnutrition in Four Cohorts of Very Old Adults, 2000–2017
Source: J Nutr Health Aging. 2022 Jul 6;26(7):706–13. doi: 10.1007/s12603-022-1820-x (PMC12876584; doi:10.1007/s12603-022-1820-x)
Supplement: Supplementary file 2 — Supplementary material, approximately 57.6 KB. [file mmc2.docx]

| **Supplementary Table 1.** Baseline characteristics of participants aged 85 years, according to cohorts | | | | | |
| --- | --- | --- | --- | --- | --- |
|  | **2000−2002 (C1)** | **2005−2007 (C2)** | **2010−2012 (C3)** | **2015−2017 (C4)** |  |
| **Characteristic** | (*n* = 137) | (*n* = 123) | (*n* = 150) | (*n* =166) | ***p*** |
| Women | 91 (66.4) | 78 (63.4) | 82 (54.7) | 101 (60.8) | 0.210 |
| Age mean (years) | 85.0 ± 0.0 | 85.7 ± 0.5 | 84.8 ± 0.4 | 85.0 ± 0.3 | <0.001 |
| Living in residential care facilities | 24 (17.5) | 25 (20.3) | 23 (15.3) | 16 (9.6) | 0.071 |
| <8 years education (*n* = 566) | 98 (71.5) | 97 (82.2) | 101 (68.7) | 87 (53.0) | <0.001 |
| Current smoker (*n* = 574) | 8 (5.9) | 5 (4.1) | 5 (3.3) | 4 (2.4) |  |
| Barthel ADL Index (0−20; *n* = 575) | 18.5 ± 3.8 | 18.0 ± 4.3 | 18.3 ± 3.8 | 18.3 ± 4.2 | 0.723 |
| Independence in P-ADL & I-ADL^a^ (*n*=573) | 50 (36.8) | 58 (47.2) | 48 (32.0) | 66 (40.2) | 0.074 |
| GDS-15 score (*n* = 554) | 3.5 ± 2.4 | 3.3 ± 2.6 | 3.3 ± 2.4 | 2.6 ± 2.2 | 0.014 |
| MMSE score (*n* = 570) | 24.5 ± 5.4 | 23.4 ± 5.6 | 23.7 ± 5.1 | 24.6 ± 5.7 | 0.172 |
| **Diagnoses** |  |  |  |  |  |
| Constipation | 37 (27.0) | 51 (41.5) | 57 (38.0) | 66 (39.8) | 0.057 |
| COPD | 17 (12.4) | 23 (18.7) | 41 (27.3) | 27 (16.3) | 0.009 |
| Dementia disorder | 27 (19.7) | 28 (22.8) | 34 (22.7) | 40 (24.1) | 0.835 |
| Depressive disorder | 32 (23.4) | 46 (37.4) | 57 (38.0) | 56 (33.7) | 0.036 |
| Diabetes mellitus | 18 (13.1) | 19 (15.4) | 29 (19.3) | 32 (19.3) | 0.416 |
| Diarrhoea | 8 (5.8) | 12 (9.8) | 15 (10.0) | 29 (17.5) | 0.011 |
| Heart failure | 25 (18.2) | 23 (18.7) | 43 (28.7) | 36 (21.7) | 0.122 |
| Hip fracture | 18 (13.1) | 13 (10.6) | 16 (10.7) | 19 (11.4) | 0.903 |
| Hypertension | 99 (72.3) | 86 (69.9) | 124 (82.7) | 136 (81.9) | 0.016 |
| Malignancy^b^ | 16 (11.7) | 13 (10.6) | 34 (22.7) | 33 (19.9) | 0.012 |
| Myocardial infarction^c^ | 3 (2.2) | 4 (3.3) | 1 (0.7) | 2 (1.2) |  |
| Parkinson's disease | 4 (2.9) | 3 (2.4) | 0 (0.0) | 3 (1.8) |  |
| Stroke | 27 (19.7) | 27 (22.0) | 33 (22.0) | 27 (16.3) | 0.547 |
| Thyroid disease | 20 (14.6) | 14 (11.4) | 35 (23.3) | 31 (18.7) | 0.052 |
| Urinary tract infection^d^ | 33 (24.1) | 30 (24.4) | 20 (13.3) | 24 (14.5) | 0.018 |
| **Drug prescriptions** |  |  |  |  |  |
| Number of drugs^e^ | 5.5 ± 3.8 | 7.7 ± 5.0 | 8.0 ± 4.3 | 7.4 ± 4.1 | <0.001 |
| Analgesics | 95 (69.3) | 88 (71.5) | 107 (71.3) | 111 (66.9) | 0.796 |
| Antidepressants | 22 (16.1) | 16 (13.0) | 26 (17.3) | 28 (16.9) | 0.774 |
| Cholinesterase inhibitors | 4 (2.9) | 3 (2.4) | 3 (2.0) | 8 (4.8) |  |
| Corticosteroids, oral | 5 (3.6) | 11 (8.9) | 3 (2.0) | 6 (3.6) |  |
| Diuretics | 57 (41.6) | 56 (45.5) | 75 (50.0) | 65 (39.2) | 0.240 |
| Drugs for acid-related symptoms | 15 (10.9) | 28 (22.8) | 40 (26.7) | 41 (24.7) | 0.006 |
| Insulin | 6 (4.4) | 5 (4.1) | 15 (10.0) | 12 (7.2) | 0.151 |
| Laxatives | 27 (19.7) | 38 (30.9) | 35 (23.3) | 35 (21.1) | 0.146 |
| Mirtazapin | 1 (0.7) | 6 (4.9) | 5 (3.3) | 3 (1.8) |  |
| Neuroleptics | 7 (5.1) | 11 (8.9) | 4 (2.7) | 4 (2.4) | 0.035 |
| Opioids | 16 (11.7) | 27 (22.0) | 16 (10.7) | 14 (8.4) | 0.005 |
| Oral antihyperglycaemics | 8 (5.8) | 15 (12.2) | 17 (11.3) | 5 (3.0) | 0.008 |
| Paracetamol | 48 (35.0) | 57 (46.2) | 71 (47.3) | 81 (48.8) | 0.077 |
| SSRIs | 19 (13.9) | 10 (8.1) | 16 (10.7) | 24 (14.5) | 0.333 |
| Vitamin B_12_ | 31 (22.6) | 48 (39.0) | 33 (22.0) | 47 (28.3) | 0.007 |
| Data are presented as mean ± standard deviation or *n* (%), unless otherwise indicated. Differences in mean values were examined using one-way analysis of variance. Differences in proportions were analysed using the chi-squared test. a. According to the ADL staircase. b. In the previous 5 years. c. In the previous year. d. At present or in the previous year. e. Regular use and *pro re nata*. ADL, activities of daily living; C, cohort; COPD, chronic obstructive pulmonary disease; GDS-15, 15-item Geriatric Depression Scale; I, instrumental; MMSE, Mini-Mental State Examination; P, personal; SSRI, selective serotonin reuptake inhibitor. | | | | | |

| **Supplementary Table 2.** Baseline characteristics of participants aged 90 years, according to cohorts | | | | | |
| --- | --- | --- | --- | --- | --- |
|  | **2000−2002 (C1)** | **2005−2007 (C2)** | **2010−2012 (C3)** | **2015−2017 (C4)** |  |
| **Characteristic** | (*n* = 129) | (*n* = 129) | (*n* = 139) | (*n* = 189) | ***p*** |
| Women | 90 (69.8) | 85 (65.9) | 90 (64.7) | 116 (61.4) | 0.488 |
| Age mean (years) | 90.0 ± 0.0 | 89.8 ± 0.4 | 89.9 ± 0.4 | 89.8 ± 0.7 | 0.010 |
| Living in residential care facilities | 58 (45.0) | 40 (31.0) | 46 (33.1) | 41 (21.7) | <0.001 |
| <8 years education (*n* = 557) | 96 (75.0) | 81 (73.0) | 95 (70.9) | 121 (65.8) | 0.311 |
| Current smoker (*n* = 584) | 4 (3.1) | 5 (3.9) | 3 (2.2) | 3 (1.6) |  |
| Barthel ADL Index (0−20) | 16.3 ± 5.3 | 17.2 ± 4.5 | 16.9 ± 4.9 | 17.2 ± 4.7 | 0.364 |
| Independence in P-ADL & I-ADL^a^ | 24 (18.6) | 34 (26.4) | 20 (14.4) | 29 (15.3) | 0.043 |
| GDS-15 score (*n* = 549) | 4.0 ± 2.8 | 3.8 ± 2.9 | 3.6 ± 2.6 | 3.1 ± 2.2 | 0.021 |
| MMSE score (*n* = 579) | 21.6 ± 7.6 | 20.6 ± 6.8 | 21.4 ± 6.8 | 21.8 ± 6.2 | 0.488 |
| **Diagnoses** |  |  |  |  |  |
| Constipation | 57 (44.2) | 64 (49.6) | 66 (47.5) | 97 (51.3) | 0.640 |
| COPD | 21 (16.3) | 18 (14.0) | 28 (20.1) | 37 (19.6) | 0.486 |
| Dementia disorder | 31 (24.0) | 45 (34.9) | 53 (38.1) | 74 (39.2) | 0.031 |
| Depressive disorder | 41 (31.8) | 57 (44.2) | 66 (47.5) | 75 (39.7) | 0.054 |
| Diabetes mellitus | 19 (14.7) | 17 (13.2) | 31 (22.3) | 32 (16.9) | 0.204 |
| Diarrhoea | 15 (11.6) | 14 (10.9) | 12 (8.6) | 44 (23.3) | <0.001 |
| Heart failure | 37 (28.7) | 39 (30.2) | 52 (37.4) | 56 (29.6) | 0.372 |
| Hip fracture | 26 (20.2) | 19 (14.7) | 28 (20.1) | 29 (15.3) | 0.457 |
| Hypertension | 67 (51.9) | 97 (75.2) | 109 (78.4) | 154 (81.5) | <0.001 |
| Malignancy^b^ | 15 (11.6) | 13 (10.1) | 14 (10.1) | 37 (19.6) | 0.028 |
| Myocardial infarction^c^ | 7 (5.4) | 4 (3.1) | 3 (2.2) | 0 (0.0) |  |
| Parkinson's disease | 2 (1.6) | 2 (1.6) | 1 (0.7) | 1 (0.5) |  |
| Stroke | 28 (21.7) | 25 (19.4) | 45 (32.4) | 36 (19.0) | 0.022 |
| Thyroid disease | 17 (13.2) | 21 (16.3) | 28 (20.1) | 39 (20.6) | 0.305 |
| Urinary tract infection^d^ | 39 (30.2) | 28 (21.7) | 27 (19.4) | 25 (13.2) | 0.003 |
| **Drug prescriptions** |  |  |  |  |  |
| Number of drugs^e^ | 7.0 ± 4.8 | 7.9 ± 4.7 | 8.7 ± 4.6 | 8.2 ± 4.2 | 0.022 |
| Analgesics | 100 (77.5) | 103 (79.8) | 111 (79.9) | 130 (68.8) | 0.053 |
| Antidepressants | 26 (20.2) | 24 (18.6) | 34 (24.5) | 38 (20.1) | 0.661 |
| Cholinesterase inhibitors | 2 (1.6) | 9 (7.0) | 2 (1.4) | 4 (2.1) |  |
| Corticosteroids, oral | 12 (9.3) | 10 (7.8) | 8 (5.8) | 15 (7.9) | 0.747 |
| Diuretics | 74 (57.4) | 57 (44.2) | 76 (54.7) | 89 (47.1) | 0.096 |
| Drugs for acid-related symptoms | 20 (15.5) | 25 (19.4) | 30 (21.6) | 47 (24.9) | 0.231 |
| Insulin | 4 (3.1) | 3 (2.3) | 13 (9.4) | 15 (7.9) |  |
| Laxatives | 49 (38.0) | 49 (38.0) | 58 (41.7) | 65 (34.4) | 0.604 |
| Mirtazapin | 1 (0.8) | 0 (0.0) | 5 (3.6) | 12 (6.3) |  |
| Neuroleptics | 10 (7.8) | 7 (5.4) | 6 (4.3) | 2 (1.1) |  |
| Opioids | 36 (27.9) | 24 (18.6) | 19 (13.7) | 23 (12.2) | 0.002 |
| Oral antihyperglycaemics | 6 (4.7) | 11 (8.5) | 16 (11.5) | 16 (8.5) | 0.248 |
| Paracetamol | 57 (44.2) | 71 (55.0) | 78 (56.1) | 105(55.6) | 0.153 |
| SSRIs | 23 (17.8) | 23 (17.8) | 26 (18.7) | 24 (12.7) | 0.421 |
| Vitamin B_12_ | 38 (29.5) | 60 (46.5) | 49 (35.3) | 56 (29.6) | 0.009 |
| Data are presented as mean ± standard deviation or *n* (%), unless otherwise indicated. Differences in mean values were examined using one-way analysis of variance. Differences in proportions were analysed using the chi-squared test. a. According to the ADL staircase. b. In the previous 5 years. c. In the previous year. d. At present or in the previous year. e. Regular use and *pro re nata*. ADL, activities of daily living; C, cohort; COPD, chronic obstructive pulmonary disease; GDS-15, 15-item Geriatric Depression Scale; I, instrumental; MMSE, Mini-Mental State Examination; P, personal; SSRI, selective serotonin reuptake inhibitor. | | | | | |

| **Supplementary Table 3.** Baseline characteristics of participants aged ≥ 95 years, according to cohorts | | | | | |
| --- | --- | --- | --- | --- | --- |
|  | **2000−2002 (C1)** | **2005−2007 (C2)** | **2010−2012 (C3)** | **2015−2017 (C4)** |  |
| **Characteristic** | (*n* = 77) | (*n* = 90) | (*n* = 120) | (*n* = 153) | ***p*** |
| Women | 62 (80.5) | 68 (75.6) | 81 (67.5) | 117 (76.5) | 0.177 |
| Age mean (years) | 96.6 ± 1.8 | 96.4 ± 2.1 | 96.0 ± 2.2 | 96.2 ± 2.0 | 0.156 |
| Living in residential care facilities | 54 (70.1) | 51 (56.7) | 63 (52.5) | 78 (51.0) | 0.038 |
| <8 years education (*n* = 401) | 61 (82.4) | 59 (78.7) | 93 (83.8) | 102 (72.3) | 0.127 |
| Current smoker (*n* = 437) | 2 (2.6) | 1 (1.1) | 1 (0.8) | 2 (1.3) |  |
| Barthel ADL Index (0−20; *n* = 438) | 12.0 ± 7.5 | 14.4 ± 5.9 | 15.2 ± 5.3 | 13.5 ± 6.4 | 0.004 |
| Independence in P-ADL & I-ADL^a^ | 5 (6.5) | 4 (4.4) | 4 (3.3) | 5 (3.3) |  |
| GDS-15 score (*n* = 383) | 4.3 ± 3.0 | 4.1 ± 2.3 | 3.8 ± 2.4 | 4.2 ± 2.9 | 0.558 |
| MMSE score (*n* = 418) | 17.3 ± 9.7 | 18.2 ± 7.0 | 19.6 ± 7.0 | 18.7 ± 7.4 | 0.240 |
| **Diagnoses** |  |  |  |  |  |
| Constipation | 45 (58.4) | 53 (58.9) | 77 (64.2) | 110 (71.9) | 0.105 |
| COPD | 10 (13.0) | 15 (16.7) | 15 (12.5) | 25 (16.3) | 0.743 |
| Dementia disorder | 34 (44.2) | 41 (45.6) | 63 (52.5) | 89 (58.2) | 0.127 |
| Depressive disorder | 20 (26.0) | 38 (42.2) | 58 (48.3) | 77 (50.3) | 0.003 |
| Diabetes mellitus | 8 (10.4) | 10 (11.1) | 20 (16.7) | 21 (13.7) | 0.547 |
| Diarrhoea | 11 (14.3) | 19 (21.1) | 26 (21.7) | 42 (27.5) | 0.151 |
| Heart failure | 23 (29.9) | 34 (37.8) | 59 (49.2) | 54 (35.3) | 0.031 |
| Hip fracture | 26 (33.8) | 22 (24.4) | 34 (28.3) | 44 (28.8) | 0.621 |
| Hypertension | 23 (29.9) | 52 (57.8) | 95 (79.2) | 114 (74.5) | <0.001 |
| Malignancy^b^ | 5 (6.5) | 5 (6.5) | 12 (10.0) | 22 (14.4) | 0.098 |
| Myocardial infarction^c^ | 2 (2.6) | 3 (3.3) | 6 (5.0) | 0 (0.0) |  |
| Parkinson's disease | 3 (3.9) | 0 (0.0) | 1 (0.8) | 1 (0.7) |  |
| Stroke | 13 (16.9) | 23 (25.6) | 25 (20.8) | 42 (27.5) | 0.273 |
| Thyroid disease | 3 (3.9) | 12 (13.3) | 26 (21.7) | 38 (24.8) |  |
| Urinary tract infection^d^ | 27 (35.1) | 34 (37.8) | 36 (30.0) | 26 (17.0) | 0.001 |
| **Drug prescription** |  |  |  |  |  |
| Number of drugs^e^ | 6.7 ± 4.4 | 9.3 ± 5.7 | 9.1 ± 4.3 | 9.2 ± 5.3 | 0.002 |
| Analgetics | 58 (75.3) | 73 (81.1) | 108 (90.0) | 127 (83.0) | 0.053 |
| Antidepressants | 8 (10.4) | 16 (17.8) | 22 (18.3) | 34 (22.2) | 0.184 |
| Cholinetserase inhibitors | 1 (1.3) | 2 (2.2) | 2 (1.7) | 2 (1.3) |  |
| Corticosteroids, oral | 1 (1.3) | 4 (4.4) | 5 (4.2) | 4 (2.6) |  |
| Diuretics | 36 (46.8) | 57 (63.3) | 79 (65.8) | 67 (43.8) | <0.001 |
| Drugs for acid-related symptoms | 7 (9.1) | 24 (26.7) | 40 (33.3) | 36 (23.5) | 0.002 |
| Insulin | 1 (1.3) | 0 (0.0) | 7 (5.8) | 10 (6.5) |  |
| laxatives | 46 (59.7) | 48 (53.3) | 62 (51.7) | 84 (54.9) | 0.729 |
| Mirtazapin | 0 (0.0) | 5 (5.6) | 3 (2.5) | 9 (5.9) |  |
| Neuroleptics | 11 (14.3) | 5 (5.6) | 3 (2.5) | 12 (7.8) |  |
| Opioids | 18 (23.4) | 21 (23.3) | 29 (24.2) | 34 (22.2) | 0.986 |
| Oral antihyperglycaemics | 5 (6.5) | 7 (7.8) | 3 (2.5) | 2 (1.3) |  |
| Paracetamol | 39 (50.6) | 55 (61.1) | 85 (70.8) | 113 (73.9) | 0.002 |
| SSRI | 5 (6.5) | 14 (15.6) | 18 (15.0) | 25 (16.3) | 0.208 |
| Vitamine B_12_ | 18 (23.4) | 38 (42.2) | 51 (42.5) | 51 (33.3) | 0.023 |
| Data are presented as mean ± standard deviation or *n* (%), unless otherwise indicated. Differences in mean values were examined using one-way analysis of variance. Differences in proportions were analysed using the chi-squared test. a. According to the ADL staircase. b. In the previous 5 years. c. In the previous year. d. At present or in the previous year. e. Regular use and *pro re nata*. ADL, activities of daily living; C, cohort; COPD, chronic obstructive pulmonary disease; GDS-15, 15-item Geriatric Depression Scale; I, instrumental; MMSE, Mini-Mental State Examination; P, personal; SSRI, selective serotonin reuptake inhibitor. | | | | | |

| **Supplementary Table 4.** Baseline characteristics of participants, excluding individuals who participated more than once | | | | | | |
| --- | --- | --- | --- | --- | --- | --- |
|  | | **2000−2002 (C1)** | **2005−2007 (C2)** | **2010−2012 (C3)** | **2015−2017 (C4)** |  |
| **Characteristic** | | (*n* = 343) | (*n* = 227) | (*n* = 279) | (*n* = 330) | ***p*** |
| Women) | | 243 (70.8) | 147 (64.8) | 166 (59.5) | 216 (65.5) | 0.032 |
| Age mean (years) | | 89.5 ± 4.5 | 89.1 ± 4.4 | 88.6 ± 4.7 | 89.0 ± 4.7 | 0.117 |
| Age range (years) | | 85−103 | 84−99 | 84−99 | 84−99 |  |
| Age group (years) | |  |  |  |  | 0.001 |
| 85 | | 137 (39.9) | 123 (54.2) | 150 (53.8) | 166 (50.3) |  |
| 90 | | 129 (37.6) | 56 (24.7) | 67 (24.0) | 83 (25.2) |  |
| ≥95 | | 77 (22.4) | 48 (21.1) | 62 (22.2) | 81 (24.5) |  |
| Living in residential care facilities | | 136 (39.7) | 69 (30.4) | 81 (29.0) | 77 (23.3) | <0.001 |
| <8 years education (*n* = 1157) | | 256 (75.3) | 171 (79.9) | 196 (71.3) | 197 (60.1) | <0.001 |
| Current smoker (*n* = 1174) | | 14 (4.1) | 8 (3.5) | 6 (2.2) | 6 (1.8) | 0.262 |
| Barthel ADL Index (0−20; *n* = 1177) | | 16.2 ± 5.9 | 17.1 ± 4.8 | 17.4 ± 4.2 | 16.9 ± 5.4 | 0.040 |
| Independence in P-ADL & I-ADL^a^ (*n*=1176) | | 79 (23.1) | 75 (33.0) | 58 (20.8) | 81 (24.7) | 0.011 |
| GDS-15 score (*n* = 1090) | | 3.8 ± 2.7 | 3.5 ± 2.5 | 3.6 ± 2.5 | 3.2 ± 2.4 | 0.021 |
| MMSE score (*n* = 1155) | | 21.8 ± 7.8 | 22.0 ± 6.4 | 22.2 ± 6.2 | 22.5 ± 6.9 | 0.683 |
| **Diagnoses** | |  |  |  |  |  |
| Constipation | | 139 (40.5) | 108 (47.6) | 126 (45.2) | 166 (50.3) | 0.076 |
| COPD | | 48 (14.0) | 42 (18.5) | 62 (22.2) | 49 (14.8) | 0.029 |
| Dementia disorder | | 92 (26.8) | 62 (27.3) | 95 (34.1) | 121 (36.7) | 0.016 |
| Depressive disorder | | 93 (27.1) | 88 (38.8) | 128 (45.9) | 131 (39.7) | <0.001 |
| Diabetes mellitus | | 45 (13.1) | 33 (14.5) | 51 (18.3) | 50 (15.2) | 0.350 |
| Diarrhea | | 34 (9.9) | 30 (13.2) | 32 (11.5) | 71 (21.5) | <0.001 |
| Heart failure | | 85 (24.8) | 64 (28.2) | 99 (35.5) | 86 (26.1) | 0.019 |
| Hip fracture | | 70 (20.4) | 38 (16.7) | 53 (19.0) | 51 (15.5) | 0.357 |
| Hypertension | | 189 (55.1) | 161 (70.9) | 226 (81.0) | 258 (78.2) | <0.001 |
| Malignancy^b^ | | 36 (10.5) | 22 (9.7) | 49 (17.6) | 56 (17.0) | 0.006 |
| Myocardial infarction^c^ | | 12 (3.5) | 8 (3.5) | 8 (2.9) | 2 (0.6) |  |
| Parkinson's disease | | 9 (2.6) | 4 (1.8) | 1 (0.4) | 3 (0.9) |  |
| Stroke | | 68 (19.8) | 42 (18.5) | 66 (23.7) | 63 (19.1) | 0.433 |
| Thyroid disease | | 40 (11.7) | 27 (11.9) | 65 (23.3) | 70 (21.2) | <0.001 |
| Urinary tract infection^d^ | | 99 (28.9) | 61 (26.9) | 49 (17.6) | 51 (15.5) | <0.001 |
| **Drug prescriptions** | |  |  |  |  |  |
| Number of drugs^e^ | | 6.4 ± 4.4 | 8.3 ± 5.4 | 8.3 ± 4.4 | 8.1 ± 4.7 | <0.001 |
| Analgesics | 253 (73.8) | | 166 (73.1) | 219 (78.5) | 234 (70.9) | 0.198 |
| Antidepressants | | 56 (16.3) | 30 (13.2) | 56 (20.1) | 64 (19.4) | 0.150 |
| Cholinesterase inhibitors | | 7 (2.0) | 4 (1.8) | 5 (1.8) | 11 (3.3) |  |
| Corticosteroids, oral | | 18 (5.2) | 17 (7.5) | 9 (3.2) | 14 (4.2) | 0.150 |
| Diuretics | | 167 (48.7) | 115 (50.7) | 159 (57.0) | 137 (41.5) | 0.002 |
| Drugs for acid-related symptoms | | 42 (12.2) | 53 (23.3) | 76 (27.2) | 77 (23.3) | <0.001 |
| Insulin | | 11 (3.2) | 6 (2.6) | 23 (8.2) | 20 (6.1) | 0.008 |
| Laxatives | | 122 (35.6) | 88 (38.8) | 90 (32.3) | 105 (31.8) | 0.300 |
| Mirtazapin | | 2 (0.6) | 7 (3.1) | 8 (2.9) | 17 (5.2) |  |
| Neuroleptics | | 28 (8.2) | 13 (5.7) | 9 (3.2) | 13 (3.9) | 0.027 |
| Opioids | | 70 (20.4) | 52 (22.9) | 41 (14.7) | 48 (14.5) | 0.020 |
| Oral antihyperglycaemics | | 19 (5.5) | 25 (11.0) | 25 (9.0) | 10 (3.0) | 0.001 |
| Paracetamol | | 144 (42.0) | 118 (52.0) | 158 (56.6) | 187 (56.7) | <0.001 |
| SSRIs | | 47 (13.7) | 24 (10.6) | 41 (14.7) | 48 (14.5) | 0.510 |
| Vitamin B_12_ | | 87 (25.4) | 95 (41.9) | 87 (31.2) | 99 (30.0) | 0.001 |
| Data are presented as mean ± standard deviation or *n* (%), unless otherwise indicated. Differences in mean values were examined using one-way analysis of variance. Differences in proportions were analysed using the chi-squared test. a. According to the ADL staircase. b. In the last 5 years. c. In the previous year. d. At present or in the previous year. e. Regular use and *pro* *re nata*. ADL, activities of daily living; P, personal; I, instrumental; GDS-15, 15-item Geriatric Depression Scale; MMSE, Mini-Mental State Examination; COPD, chronic obstructive pulmonary disease; SSRI, selective serotonin reuptake inhibitor | | | | | | |

| **Supplementary Table 5**. Differences in BMI among cohorts, excluding individuals who participated more than once | | | | | | | | | | |
| --- | --- | --- | --- | --- | --- | --- | --- | --- | --- | --- |
|  |  | | **2000−2002 (C1)** | | | **2005−2007 (C2)** | | **2010−2012 (C3)** | **2015−2017 (C4)** |  |
|  |  | | (*n* = 343) | | | (*n* = 227) | | (*n* = 279) | (*n* = 330) | ***p*** |
| **Whole sample** | | |  | | |  | |  |  |  |
| Mean BMI | | 24.8 ± 4.7 | | 25.3 ± 4.1 | | | 25.7 ± 4.3 | | 26.4 ± 4.8^a,b^ | <0.001 |
| BMI categ. | |  | |  | | |  | |  | 0.001 |
| <18.5 | | 26 (7.6) | | 8 (3.5) | | | 11 (3.9) | | 6 (1.8) |  |
| 18.5−24.9 | | 173 (50.4) | | 108 (47.6) | | | 120 (43.0) | | 134 (40.6) |  |
| 25.0−29.9 | | 98 (28.6) | | 78 (34.4) | | | 102 (36.6) | | 124 (37.6) |  |
| ≥30.0 | | 46 (13.4) | | 33 (14.5) | | | 46 (16.5) | | 66 (20.0) |  |
| **85 years** | | |  | | |  | |  |  |  |
| Mean BMI | | 25.6 ± 4.2 | | | 25.7 ± 4.0 | | 26.2 ± 4.4 | | 27.4 ± 4.7^a,b^ | 0.001 |
| BMI categ. | |  | | |  | |  | |  | 0.112 |
| <18.5 | | 4 (2.9) | | | 3 (2.4) | | 4 (2.7) | | 2 (1.2) |  |
| 18.5−24.9 | | 72 (52.6) | | | 55 (44.7) | | 63 (42.0) | | 57 (34.3) |  |
| 25.0−29.9 | | 44 (32.1) | | | 44 (35.8) | | 54 (36.0) | | 67 (34,3) |  |
| ≥30.0 | | 17 (12.4) | | | 21 (17.1) | | 29 (19.3) | | 40 (24.1) |  |
| **90 years** | | |  | | |  | |  |  |  |
| Mean BMI | | 25.0 ± 4.9 | | | 25.2 ± 4.1 | | 24.9 ± 4.3 | | 25.9 ± 4.5 | 0.496^c^ |
| BMI categ. | |  | | |  | |  | |  | 0.480 |
| <18.5 | | 11 (8.5) | | | 1 (1.8) | | 4 (6.0) | | 1 (1.2) |  |
| 18.5−24.9 | | 53 (41.1) | | | 29 (51.8) | | 30 (44.8) | | 37 (44.6) |  |
| 25.0−29.9 | | 42 (32.6) | | | 18 (32.1) | | 23 (34.3) | | 30 (36.1) |  |
| ≥30.0 | | 23 (17.8) | | | 8 (14.3) | | 10 (14.9) | | 15 (18.1) |  |
| **≥ 95 years** | | |  | | |  | |  |  |  |
| Mean BMI | | 23.0 ± 4.5 | | | 24.1 ± 4.0 | | 25.4 ± 3.8^a^ | | 25.0 ± 4.8^a^ | 0.006 |
| BMI categ. | |  | | |  | |  | |  | 0.027 |
| <18.5 | | 11 (14.3) | | | 4 (8.3) | | 3 (4.8) | | 3 (3.7) |  |
| 18.5−24.9 | | 48 (62.3) | | | 24 (50.0) | | 27 (43.5) | | 40 (49.4) |  |
| 25.0−29.9 | | 12 (15.6) | | | 16 (33.3) | | 25 (40.3) | | 27 (33.3) |  |
| ≥30.0 | | 6 (7.8) | | | 4 (8.3) | | 7 (11.3) | | 11 (13.6) |  |
| Data are presented as mean ± standard deviation or *n* (%). Differences in mean values were examined using one-way analysis of variance with Bonferroni correction. Differences in proportions were analysed using chi–squared tests. Post-hoc tests: a. significant difference vs. C1; b. significant difference vs. C2; c. no significant difference. BMI, body mass index (kg/m^2^). | | | | | | | | | | |

| **Supplementary Table 6.** Differences in MNA score among cohorts, excluding individuals who participated more than once | | | | | |
| --- | --- | --- | --- | --- | --- |
|  | **2000−2002 (C1)** | **2005−2007 (C2)** | **2010−2012 (C3)** | **2015−2017 (C4)** |  |
|  | (*n* = 343) | (*n* = 227) | (*n* = 279) | (*n* = 330) | ***p*** |
| **Whole sample** | |  |  |  |  |
| Mean MNA score | 23.2 ± 4.7 | 23.9 ± 3.9 | 24.5 ± 3.4^a^ | 23.7 ± 4.0 | 0.001 |
| MNA score categ. | |  |  |  | 0.001 |
| <17 | 42 (12.2) | 12 (5.3) | 9 (3.2) | 25 (7.6) |  |
| 17.0−23.5 | 109 (31.8) | 80 (35.2) | 83 (29.7) | 113 (34.2) |  |
| 24−30 | 192 (56.0) | 135 (59.5) | 187 (67.0) | 192 (58.2) |  |
| **85 years** | |  |  |  |  |
| Mean MNA score | 24.9 ± 3.2 | 24.8 ± 3.5 | 25.0 ± 3.2 | 24.9 ± 3.5 | 0.991^c^ |
| MNA score categ. | |  |  |  | 0.792 |
| <17 | 5 (3.6) | 3 (2.4) | 3 (2.0) | 7 (4.2) |  |
| 17.0−23.5 | 35 (25.5) | 34 (27.6) | 44 (29.3) | 38 (22.9) |  |
| 24−30 | 97 (70.8) | 86 (69.9) | 103 (68.7) | 121 (72.9) |  |
| **90 years** | |  |  |  |  |
| Mean MNA score | 22.8 ± 4.6 | 23.6 ± 3.5 | 24.4 ± 3.6 | 23.8 ± 3.7 | 0.057^c^ |
| MNA score categ. | |  |  |  | 0.016 |
| <17 | 16 (12.4) | 2 (3.6) | 2 (3.0) | 3 (3.6) |  |
| 17.0−23.5 | 46 (35.7) | 23 (41.1) | 17 (25.4) | 34 (41.0) |  |
| 24−30 | 67 (51.9) | 31 (55.4) | 48 (71.6) | 46 (55.4) |  |
| **≥ 95 years** | |  |  |  |  |
| Mean MNA score | 20.6 ± 5.7 | 21.8 ± 4.2 | 23.5 ± 3.4^a^ | 21.0 ± 4.2^b^ | 0.001 |
| MNA score categ. | |  |  |  | 0.004 |
| <17 | 21 (27.3) | 7 (14.6) | 4 (6.5) | 15 (18.5) |  |
| 17.0−23.5 | 28 (36.4) | 23 (47.9) | 22 (35.5) | 41 (50.6) |  |
| 24−30 | 28 (36.4) | 18 (37.5) | 36 (58.1) | 25 (30.9) |  |
| Data are presented as mean ± standard deviation or *n* (%). Differences in mean values were examined using one-way analysis of variance with Bonferroni correction. Differences in proportions were analysed using chi–squared tests. Post-hoc tests: a. significant difference vs. C1; b. significant difference vs. C3; c. no significant difference. MNA, Mini Nutritional Assessment. | | | | | |
